# Association between *ACE* (rs4646994), *FABP2* (rs1799883), *MTHFR* (rs1801133), *FTO* (rs9939609) Genes Polymorphism and Type 2 Diabetes with Dyslipidemia

**DOI:** 10.22088/acadpub.BUMS.6.2.6

**Published:** 2017-07-04

**Authors:** Syed Tasleem Raza, Shania Abbas, Zeba Siddiqi, Farzana Mahdi

**Affiliations:** 1 *Department of Biochemistry, Era’s Lucknow Medical College and Hospital, Lucknow, India.*; 2 *Department of Medicine Era’s Lucknow Medical College and Hospital, Lucknow, India.*

**Keywords:** Genetic polymorphisms, type 2 diabetes mellitus (T2DM), Dyslipidemia

## Abstract

Diabetic dyslipidemia is one of the leading causes of coronary artery disease (CAD) death. Genetic and environmental factors play an important role in the development of type 2 diabetes mellitus (T2DM) and dyslipidemia. The present study was aimed to investigate the association of *ACE *(rs4646994),* FABP2 *(rs1799883), *MTHFR *(rs1801133) and* FTO *(rs9939609) genes polymorphism in T2DM with dyslipidemia. Totally, 559 subjects including 221 T2DM cases with dyslipidemia, 158 T2DM without dyslipidemia and 180 controls were enrolled. *ACE* genes polymorphism was evaluated by polymerase chain reaction (PCR), while *MTHFR*, *FABP2*, *FTO* genes polymorphisms were evaluated by PCR and restriction fragment length polymorphism (RFLP). Significant association of *ACE* and *MTHFR* genes polymorphisms were found in both group of cases [T2DM with dyslipidemia (P<0.001, and P=0.008, respectively) and T2DM without dyslipidemia (P=0.003, and P=0.010, respectively)] while *FABP2* and *FTO* genes polymorphisms were significantly associated with T2DM without dyslipidemia (P=0.038, and P= 0.019, respectively). This study concludes that *ACE*, *FABP2*, *FTO* and *MTHFR* genes are associated with T2DM. Additionally, it also seems that *ACE* and *MTHFR* genes might be further associated with the development of dyslipidemia in T2DM cases.

Type 2 diabetes mellitus (T2DM) is the most frequent subtype of diabetes characterized by high sugar (glucose) levels in blood resulting from defects in insulin secretion and/or insulin action. Hyperglycemia is associated with long-term problems like failure of different organs, especially the eyes, kidneys, nerves, heart, and blood vessels. It could lead to lipid abnormalities in patients with diabetes. Dyslipidemia encompasses high plasma triglyceride concentration, decreased high- density lipoprotein (HDL) cholesterol concentration and increased concentration of low-density lipoprotein (LDL) cholesterol particles. In recent decades, there has been an unusual rise in the prevalence of T2DM, and it is predicted that the number of people with T2DM will increase from 350 million to 592 million by 2035 (1). Dyslipidemia is an established risk factor for cardiovascular diseases (CVD) in T2DM (2). It is a multifactorial disorder caused by genetic and environmental factors. Genetic linkage analysis and candidate-gene approach have implicated several loci and candidate genes like apolipoprotein E, IL-6, lipoprotein lipase for predisposition to dyslipidemia (3-6).

The renin-angiotensin system (RAS) is not only involved in cardiovascular haemodynamics, but also plays an essential role in the development of CVD. Angiotensin-converting enzyme (ACE), a key factor in the RAS, catalyzes the conversion of angiotensin I to angiotensin II in the liver and inactivates bradykinin in many tissues. Activation of the RAS through insulin resistance may promote the development of dyslipidemia and diabetes. MTHFR catalyzes the conversion of 5, 10-methylenetetrahydrofolate to 5-methyl-tetrahy-drofolate, and is an important enzyme for the homocysteine metabolic pathway. Previous studies have found the association of *MTHFR* C677T allele in DNA hypomethylation, which is further associated with metabolic syndrome (MetS) and its components (7, 8). MetS is characterized by various metabolic abnormalities, including central obesity, dyslipidemia, elevated blood pressure (BP) and high glucose concentrations. Several studies have found possible association of Ala54Thr polymorphism of the fatty acid binding protein 2 (*FABP2*) gene with insulin resistance, dyslipidemia and obesity (9-11). The aim of this study was to determine the associations between the mentioned gene polymorphisms (*ACE*, *MTHFR*, *FABP2*), and T2DM with dyslipidemia.

## Materials and methods

**Subjects**

A written informed consent was taken from all participants before collecting their blood samples. Eventually, blood samples of 559 subjects including (221 T2DM cases with dyslipidemia, 158 T2DM without dyslipidemia and 180 controls) were collected from the Department of Medicine of Era’s Lucknow Medical College & Hospital, Lucknow. The following detailed information of each patient was obtained such as: age, alcohol consumption, body mass index (BMI), height, weight, cigarette smoking, family history etc. T2DM cases were defined as patients with a fasting blood sugar (FBS) level of more than 6.99 mmol/l. Dyslipidemia was considered present when one or more lipid values {total cholesterol, LDL and triglycerides (TG)} increased or decreased HDL alone or in combination, using as cutoff values those recommended by the ATP III guidelines on dyslipidemia and atherosclerosis prevention (12). Samples having a FBS level below 6.11 mmol/l without family history of diabetes were included in the study as controls.

Protocol and procedures employed were reviewed and approved by the institutional ethical review committee.

**Biochemical estimations**

BMI was calculated according to quetelet equation by using weight in kilograms/height in meter square. Serum creatinine concentration was assessed by a kinetic Jaffe method. FBS and random blood sugar (glucose oxidase-peroxidase method), serum cholesterol (cholesterol oxidase-peroxidase), serum triglyceride (glycerol phosphate oxidase-peroxidase-amidopyrine method), HDL cholesterol (immunoinhibition) were assessed by XL-300 Transasia fully automated-analyzer Transasia, Mannheim, Germany. Very low-density lipoprotein (VLDL) was determined by enzymatic method. LDL cholesterol levels were calculated by using the Friedewald formula. HbA1C was measured using semi-autoanalyzer (Transasia, Mannheim, Germany).

**DNA extraction**

Five milliliters of peripheral blood was collected from all the subjects in 0.5 M EDTA tubes. Genomic DNA was isolated from whole blood using the standard phenol-chloroform extraction method (13). The DNA concentration was determined by Nanodrop and stored at -20 C.

**Analysis of polymorphisms**

***ACE polymorphism***

**Fig.1:**
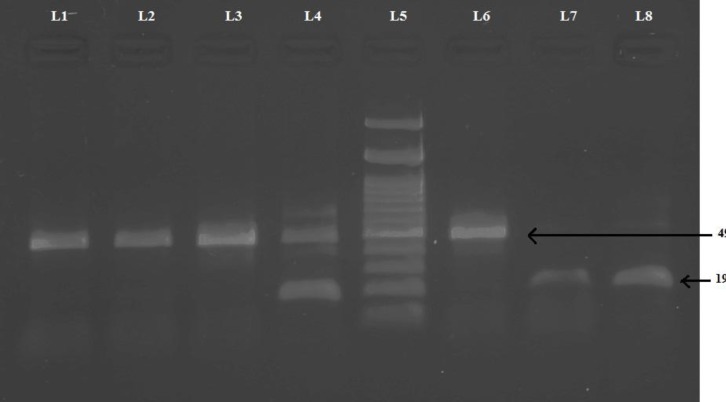
ACE gene polymorphism. Agarose gel representing different genotypes: L7, 8: DD genotype; L4: ID genotype; L1, 2, 3, 6: II genotype; L5: 100 bp ladder

PCR was employed for genotyping of the *ACE* I/D polymorphism (rs4646994). Reactions were performed with 10 pmol of each primer (14): forward primer 5’-CTG GAG ACC ACT CCC ATC CTT TCT-3’, reverse primer 5’-GAT GTG GCC ATC TTC GTC AGA T -3’, in a final volume of 20 μl containing 3 mM MgCl2, 50 mM KCl, 10 mM Tris-HCl (pH 8.4), 0.5 mM of each dNTP and 2 U Taq polymerase. PCR amplification was carried out after initial denaturation at 94^ o^C for 5 min, followed by 35 cycles of denaturation at 94^ o^C for 45 s, annealing at 60^ o^C for 1 min and 15 s, extension at 72^ o^C for 2 min and 30 s and final ex-tension at 72^ o^C for 5 min. PCR products were separated on 2% ethidium bromide stained agarose gel and visualized by UVP BIOIMAGING gel doc system. The product was 490 bp for allele I and 190 bp for allele D (Figure 1).

**Fig 2:**
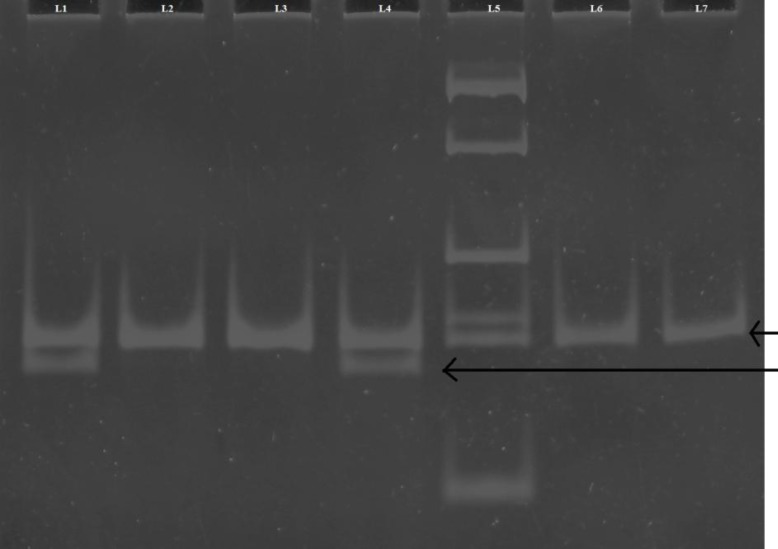
*MTHFR* gene polymorphism. Polyacrylamide gel picture showing PCR-RFLP products of *MTHFR* gene. L2, 3, 6: CC (-/-) genotype; L1, 4: CT (+/-) genotype; L7: undigested PCR product; L5: 100 bp ladder

**Fig 3:**
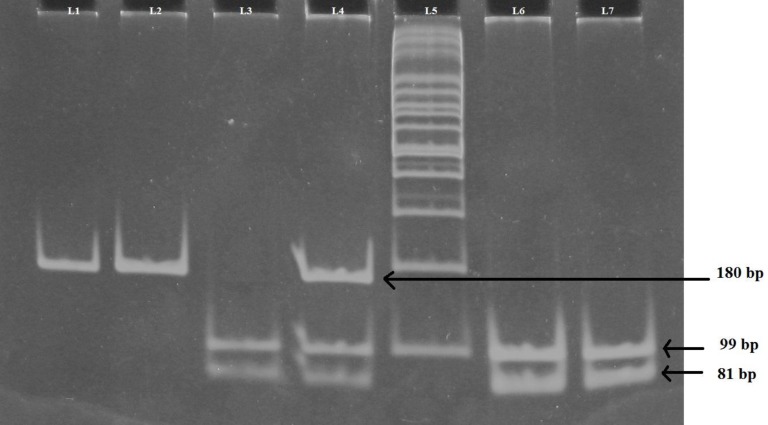
*FABP2* gene polymorphism. Polyacrylamide gel picture showing digested PCR products for *FABP2* gene. L4: AT genotype; L3, 6, 7: TT genotype; L5: 100 bp ladder; L2: AA genotype; L1: undigested PCR product (180 bp

**Fig 4:**
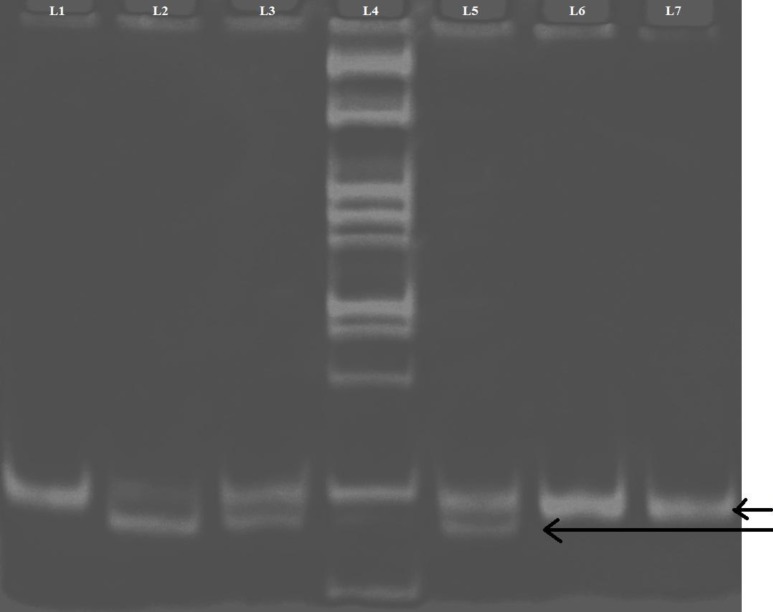
*FTO* gene polymorphism. Polyacrylamide gel picture showing digested PCR products for *FTO* gene. L2, 3, 5: AT genotype (182 and 154 bp); L6, 7: TT genotype (182 bp); L4: 100 bp ladder; L1: undigested PCR product of *FTO* (182 bp

***MTHFR polymorphism***

*MTHFR* genotyping (rs1801133) was performed by PCR and restriction fragment length polymorphism. Reactions were performed with 10 pmol of each primer (15): forward primer 5’-TGA AGG AGA AGG TGT CTG CGG GA-3’, reverse primer 5’-AGG ACG GTG CGG TGA GAG TG-3’, in a final volume of 20 μl containing 3 mM MgCl2, 50 mM KCl, 10 mM Tris-HCl (pH 8.4), 0.5 mM of each dNTP and 2 U Taq polymerase. PCR amplification was carried out following initial denaturation at 94 °C for 4 min followed by 35 cycles of 94 °C for 30 s, 61 °C for 30 s, 72 °C for 30 s. The final extension was carried out at 72 °C for 7 min. The presence of product was verified on a 2% agarose gel stained with ethidium bromide. PCR products were digested with HinfI (New England Biolabs) in a 15 µl final volume, which contained 10 µl of PCR product, 1x NE Buffer, 10 U HinfI, and was further incubated overnight at 37 °C. The digested product was separated on a 10% polyacrylamide gel stained by ethidium bromide and visualized under UV light illumination (Figure2).

***FABP2 polymorphism***

*FABP2* genotyping (rs1799883) was performed by PCR and restriction fragment length polymorphism. Reactions were performed with 10 pmol of each primer (16): forward primer 5’-ACAGGTGTTAATATAGTGAAAAG-3’ and reverse primer 5’-TACCCTGAGTTCAGTTCCG-TC-3’, in a final volume of 20 µl containing 0.3 U of Taq DNA polymerase, 10 mmol/l Tris-HCl pH 8.3, 50 mmol/l of KCl, 1.5 mmol/l of MgCl2, 100 mmol/l of dNTPs. PCR amplification was carried out under the following conditions: 35 cycles for 1 min at 94 °C, 1 min at 58 °C and 1 min at 72 °C. The PCR products were analyzed on 2% agarose gel stained with ethidium bromide to certify the proper amplification. The amplified PCR products of 180 bp were digested with the addition of 2 U HhaI (New England Biolabs), 10 mmol/l Tris-HCl pH 7.9, 50 mmol/l NaCl, 10 mmol/l MgCl2 and 1 mmol/l dithiothreitol. After incubation at 37 °C for 2 hours, digested samples were separated on 10% ethidium bromide stained polyacrylamide gel after electrophoresis and were visualized by UVP BIOIMAGING gel doc system (Figure 3). 

***FTO polymorphism***

*FTO* genotyping (rs9939609) was performed by PCR and restriction fragment length polymorphism. Reactions were performed with 10 pmol of each primer (17): forward primer 5’-AAC TGG CTC TTG AAT GAA ATA GGA TTCAGA-3’ and reverse primer 5’-AGAGTAACAGAGA-CTATCCAAGTGCAGTAC-3’, in a final volume of 20 µl containing 0.3 U of Taq DNA polymerase, 10 mmol/l Tris-HCl pH 8.3, 50 mmol/l of KCl, 1.5 mmol/l of MgCl2, 100 mmol/l of dNTPs. PCR amplification was carried out following initial denaturation at 94 ^o^C for 5 min followed by 20 cycles of 94 ^o^C for 45 s, 61 ^o^C for 4 5s (dropping 0.5 ^o^C per cycle), and 72 ^o^C for 45 s. After this, the PCR mix was amplified for 15 cycles following 94 ^o^C for 45 s, 51 ^o^C for 45 s and 72 ^o^C for 45 s, and then a final extension was performed at 72 ^o^C for 10 min. Thus, PCR products obtained were incubated at 37 ^o^C for 16 h with 2 U ScaI (New England, Biolabs). Upon running the final products on a 10% ethidium bromide stained polyacrylamide gel, the T allele produced a 182 bp band and the A allele produced 154 and 28 bp bands (Figure 4).

**Statistical analysis**

The sample size was calculated (calculations were based on the proportion of *ACE* genotypes among the cases and controls) using the following formula:

N= (Zα+Zβ)2/{ln(1−e)}∗[{(1−p1)/p1}+(1−p2)/p2}]

where N is the required sample size for one group (when the groups are equal), Z is the level of significance, p1 = 0.576 and p2 = 0.656 (proportion of *ACE* gene (I/D) polymorphism in cases and controls) (18). Based on a 95% level of significance and 80% expected power (18.2% type 2 error), the minimum sample size was 135 in each group. Based on the availability of cases, when the number of subjects increases, the power will increase correspondingly. All the statistical analysis was performed with SPSS (statistical package for the social sciences) version 12 software. Clinical data are expressed as mean ± SD. Chi-square test was used for the comparison of genotyping data between cases and controls. P-values ≤0.05 were considered as significant. Odds ratios (OR) and 95% confidence intervals (CI) were calculated to test the relative risk for association. Other variables were compared using Student's t-test for normally-distributed variables.

## Results

Our study included 559 subjects, 221 T2DM cases with dyslipidemia, 158 T2DM without dyslipidemia and 180 controls. The mean age, clinical and biochemical parameters of cases and controls are shown in Table 1. Significant association of mean BMI, TG, HDL, VLDL were found in T2DM with dyslipidemia compared to T2DM without dyslipidemia and healthy controls (P<0.001). The genotypes and alleles frequency of *ACE, MTHFR, FABP2,* and *FTO* are shown in Table 2. Significant difference was observed in the frequency of *ACE* II, ID genotypes when comparing T2DM without dyslipidemia with controls (P<0.001, P=0.007) and T2DM with dyslipidemia (P<0.001, P<0.001). The frequency of *MTHFR* CC genotype was significantly lower and *MTHFR* CT genotype was significantly higher in T2DM with and without dyslipidemia compared to the controls (P<0.001). *FABP2* gene was significantly associated with T2DM without dyslipidemia compared to the controls (P<0.001). The frequency of *FABP2* AA genotype was higher in T2DM with dyslipidemia cases compared to T2DM without dyslipidemia cases and the frequency of *FABP2* AT genotype was significantly lower in T2DM cases with dyslipidemia compared to T2DM cases without dyslipidemia (P<0.001). Significant association of *FTO* AA genotype was observed in T2DM cases without dyslipidemia compared to the controls (P<0.001).

## Discussion

T2DM is considered to be a complex disorder which results from the interaction of genes and environment factors. To date, various mutations have been linked to T2DM risk (3-6). In our study,* ACE*, *FABP2*, *FTO *and *MTHFR* genotype distributions in all cases and controls were in line with Hardy-Weinberg equilibrium (all P>0.05, data not shown).

Studies throughout all the major ethnic groups have shown highly inconsistent findings for the association of *ACE I/D* polymorphism with the risk of T2DM and its complications. Previous studies conducted in Tunisian, Indian, and Iranian populations have found *ACE* D allele to be more common in T2DM and related complications (19, 20); while the studies conducted in Malays and Indonesians have found no association of either allele with T2DM or related cardiovascular and renal diseases (21, 22). In our study, the frequency of *ACE* II genotype was 35.4% in T2DM without dyslipidemia cases which is significantly higher in comparison with 19.1% in Brazilian and 10.8% in UAE population (23, 24). The frequency of *ACE* ID genotype was 65.2% in T2DM cases with dyslipidemia c which is significantly higher in comparison to the Kuwaiti T2DM cases with cardiovascular disease 44.1% (24). Xu et al. have found significant association of *ACE* ID polymorphism with T2DM, regardless of the absence or presence of dyslipidemia (P<0.001) (25), and our study showed similar results (P<0.001).

**Table 1:** Clinical and biochemical parameters of type 2 diabetes mellitus with and without dyslipidemia cases and controls

	**Control** **(N= 180)**	**T2DM without dyslipidemia ** **(N= 158)**	**T2DM with dyslipidemia ** **(N= 221)**
Age (years)	43.8 ± 10.5	44.7 ± 8.9	46.6 ± 11.1
Body mass index (kg/m^2^)	23.0 ± 2.6	24.8 ± 2.6	26.1 ± 2.5
Random blood sugar (mmol/l)	6.6 ± 2.5	11.5 ± 5.8	12.6 ± 7.3
Serum creatinine (µmol/l)	1070 ± 475	1130 ± 976	1280 ± 998
Glycated hemoglobin (%)	5.6 ± 0.3	7.8 ± 0.5	8.1 ± 0.9
Serum cholesterol (mmol/l)	9.1 ± 1.2	9.8 ± 2.4	10.1 ± 2.9
Triglyceride (mmol/l)	1.2(0.86-0.45)	1.7(1.3-2.45)	1.9(1.2-2.49)
High-density lipoprotein (mmol/l)	2.2 ± 0.6	2.9 ± 0.8	3.2 ± 0.9
Very low-density lipoprotein (mmol/l)	1.7 ± 0.9	1.9 ± 1.4	2.6 ± 1.2
Low-density lipoprotein (mmol/l)	5.0 ± 0.9	4.8 ± 1.6	5.2 ± 1.9

**Table 2:** The genotype, and allele frequencies of *ACE, MTHFR, FABP-2 *and *FTO* genes and their statistical analysis among T2DM with dyslipidemia, T2DM without dyslipidemia cases and controls

		**T2DM without Dyslipidemia (158)**	**T2DM with Dyslipidemia (221)**	**Control (180)**	**T2DM without Dyslipidemia (158)/T2DM with Dyslipidemia (221)**	**T2DM without Dyslipidemia (158)/Control (180)**	**T2DM with Dyslipidemia (221)/Control(180)**
*ACE*		N (Frequency)	N (Frequency)	N (Frequency)	p values	p values	p values
Genotype	II	56 (35.4%)	44 (19.9%)	25 (13.9%)	<0.001	<0.001	0.112
	ID	69 (43.7%)	144 (65.2%)	105 (58.3%)	<0.001	0.007	0.161
	DD	33 (20.9%)	33 (14.9%)	50 (27.8%)	0.132	0.142	0.002
Allele	I	181(57.3%)	232 (52.5%)	155 (43.1%)	0.192	<0.001	0.008
	D	135 (42.7%)	210 (47.5%)	205 (56.9%)	0.192	<0.001	0.008
***FABP2***							
Genotype	AA	13 (8.2%)	56 (25.4%)	40 (22.2%)	<0.001	<0.001	0.467
	AT	119 (75.3%)	127 (57.4%)	114 (63.3%)	<0.001	0.017	0.233
	TT	26 (16.5%)	38 (17.2%)	26 (14.5%)	0.85	0.609	0.455
Allele	A	145 (45.9%)	239 (54.1%)	194 (53.9%)	0.026	0.038	0.958
	T	171 (54.1%)	203 (45.9%)	166 (46.1%)	0.026	0.038	0.958
***FTO***							
Genotype	AA	15 (9.5%)	4 (1.8%)	0 (NA)	<0.001	<0.001	0.070
	AT	113 (71.6%)	168 (76.0%)	131 (72.8%)	0.324	0.797	0.459
	TT	30 (18.9%)	49 (22.2%)	49 (27.2%)	0.452	0.074	0.242
Allele	A	143 (45.2%)	176 (39.8%)	131 (36.4%)	0.135	0.019	0.320
	T	173 (54.8%)	266 (60.2%)	229 (63.6%)	0.135	0.019	0.320
***MTHFR***							
Genotype	CC	58 (36.7%)	94 (42.5%)	102 (56.7%)	0.25	<0.001	0.005
	CT	74 (46.8%)	88 (39.8%)	52 (28.9%)	0.173	<0.001	0.022
	TT	26 (16.5%)	39 (17.7%)	26 (14.4%)	0.762	0.609	0.387

According to several epidemiological studies, carriers of the *MTHFR* C677T or 677TT genotype were found to be more susceptible to developing hypertension, and dyslipidemia which are important factors in the identification of metabolic syndrome (26, 27). The frequency of *MTHFR* CC genotype was significantly lower in T2DM cases with and without dyslipidemia compared to the controls, while the frequency of *MTHFR* CT genotype was significantly higher in T2DM cases with and without dyslipidemia compared to the controls (P<0.001). The frequency of *MTHFR* CC genotype in T2DM with dyslipidemia cases was 42.5% which is higher in comparison with Chinese hypertensive dyslipidemia cases 25.4% (28). Errera et al. have demonstrated that the frequencies of *MTHFR* C and T allele were 68% and 32% in Brazilian T2DM cases (29). Similar results were obtained in our study where the C and T allele frequencies were 60.1% and 39.9%, respectively in T2DM cases. The frequency of *MTHFR* CC genotype was 36.71% in T2DM cases without dyslipidemia which is higher in comparison with Turkish T2DM cases (29%) (30).

Studies have failed to demonstrate the association of *FABP2* gene with T2DM in Asian Indians (10, 11). Study conducted by Guettier et al. in South Indian population, showed that *FABP2* genetic polymorphisms where neither associated with metabolic syndrome in patients with diabetes nor with dyslipidemia (P=0.682) (31). In our population, *FABP2* gene was significantly associated with T2DM cases without dyslipidemia compared to the controls (P<0.001). The frequency of *FABP2* AA genotype was higher in T2DM cases with dyslipidemia compared to T2DM cases without dyslipidemia and the frequency of *FABP2* AT genotype was significantly lower in T2DM cases with dyslipidemia compared to T2DM cases without dyslipidemia (P<0.001). We have observed that the frequency of *FABP2* AA genotype was 25.3% in T2DM without dyslipidemia cases which is similar to Asians (27%), while lower in comparison with Caucasians (57%) (32, 33). The frequency of *FABP2* AT genotype was 57.47% in T2DM cases without dyslipidemia which is significantly higher in comparison with Mexican-American (41.9%) and Saudi (38%) populations (34, 35). The frequency of TT genotype was 17.2% in T2DM cases without dyslipidemia which is significantly higher in comparison with Caucasians (9%) (36).

A significant association of *FTO* AA genotype was observed in T2DM cases without dyslipidemia compared to the controls (P<0.001). Legry et al. found that the homozygous AA genotype of *FTO* gene was associated with a higher risk of T2DM (P=02) (37). Similar results were found in our study (P<0.001). The frequency of *FTO* AT genotype was 71.5% in T2DM cases without dyslipidemia which is significantly higher in comparison with Chinese (47.6%) and American (47.3%) populations (38, 39). The frequency of *FTO* TT genotype was 18.9% in T2DM cases without dyslipidemia which is significantly lower in comparison with Chinese T2DM cases (45%) (40).

In conclusion* ACE*, *FABP2*, *FTO* and *MTHFR* genes were found to be associated with T2DM, and additionally *ACE* and *MTHFR* genes might be implicated with the development of dyslipidemia in T2DM cases. Further analysis with larger sample size is required to validate this study.
